# Task-Relevant Information Modulates Primary Motor Cortex Activity Before Movement Onset

**DOI:** 10.3389/fnhum.2018.00093

**Published:** 2018-03-14

**Authors:** Cristian B. Calderon, Filip Van Opstal, Philippe Peigneux, Tom Verguts, Wim Gevers

**Affiliations:** ^1^Centre for Research in Cognition and Neurosciences (CRCN), ULB Neuroscience Institute, Faculté de Psychologie et Sciences de l’Éducation, Université Libre de Bruxelles, Brussels, Belgium; ^2^Department of Experimental Psychology, Ghent University, Ghent, Belgium; ^3^Department of Psychology, University of Amsterdam, Amsterdam, Netherlands; ^4^UR2NF—Neuropsychology and Functional Neuroimaging Research Unit at CRCN, Brussels, Belgium

**Keywords:** action planning, primary motor cortex, affordance competition hypothesis, fMRI

## Abstract

Monkey neurophysiology research supports the affordance competition hypothesis (ACH) proposing that cognitive information useful for action selection is integrated in sensorimotor areas. In this view, action selection would emerge from the simultaneous representation of competing action plans, in parallel biased by relevant task factors. This biased competition would take place up to primary motor cortex (M1). Although ACH is plausible in environments affording choices between actions, its relevance for human decision making is less clear. To address this issue, we designed an functional magnetic resonance imaging (fMRI) experiment modeled after monkey neurophysiology studies in which human participants processed cues conveying predictive information about upcoming button presses. Our results demonstrate that, as predicted by the ACH, predictive information (i.e., the relevant task factor) biases activity of primary motor regions. Specifically, first, activity before movement onset in contralateral M1 increases as the competition is biased in favor of a specific button press relative to activity in ipsilateral M1. Second, motor regions were more tightly coupled with fronto-parietal regions when competition between potential actions was high, again suggesting that motor regions are also part of the biased competition network. Our findings support the idea that action planning dynamics as proposed in the ACH are valid both in human and non-human primates.

## Introduction

A recent embodied view on decision-making (Cisek and Pastor-Bernier, 2014), based on neurophysiological recordings in non-human primates (Shadlen et al., 2008), suggests that decision-making arises from sensorimotor areas. This evolutionary perspective hinges on the idea that neural systems have evolved within environments affording ever changing potential actions, and was formalized in the affordance competition hypothesis (ACH; Cisek, 2007). This hypothesis proposes that task-relevant information pertinent for selecting an action modulates the competition between simultaneous action plans in the parietal reach region (PRR; Klaes et al., 2011), the dorsal premotor cortex (PMd; Cisek and Kalaska, 2005), and up to the primary motor cortex (M1; Coles et al., 1985; Bastian et al., 2003; Michelet et al., 2010). Relevant information can be action probability (Calderon et al., 2015) or reward (Klein-Flügge and Bestmann, 2012), amongst others. Once activity in favor of a specific action plan reaches a threshold, the decision is made and implemented (Gold and Shadlen, 2007; Thura et al., 2012). Thus, potential responses would be active in motor cortex before movement onset.

While the idea of task-relevant information biasing action plans in sensorimotor regions has been validated in species and contexts principally affording choices between concrete actions, it remains to be verified in humans, who have a higher capacity for making abstract choices. As a consequence of this capacity, it was proposed that the human action selection system has evolved to be segregated from sensorimotor areas executing the decision output even in the case of simple visually instructed action selection. For instance, the seminal studies of Heekeren et al. (2004, 2008) suggest that cognitive information is integrated in the dorsolateral prefrontal cortex (DLPFC). Therefore, the possibility remains that the level at which task-relevant information is integrated to make a decision marks a phylogenetic breakpoint, and is fundamentally different in monkeys (up to M1) than in humans (in (pre)frontal areas; Rorie and Newsome, 2005).

Task-relevant information leaking up to the motor cortex before movement onset has gained support in humans from Transcranial Magnetic Stimulation (TMS) studies showing, for instance, that cortico-spinal excitability (CSE) before movement onset correlates with the amount and probability of reward associated with the chosen action (e.g., Gupta and Aron, 2011; Klein-Flügge and Bestmann, 2012). Yet, it remains unclear whether one can selectively target specific cortical areas by TMS (Bolognini and Ro, 2010); in other words, TMS results may derive from activation of neighboring premotor areas. It is therefore important to find converging evidence using a more spatially precise methodology on whether human M1 activity is biased by task-relevant information prior to making a decision. Based on neurophysiological and TMS studies discussed above, we thus first predict to find signals reflecting task-relevant information in M1 activity before movement onset.

Second, to investigate the hypothesis that M1 activity prior to making a decision reflects action planning, we tested whether M1 areas were coupled with other brain areas that have been shown to be consistently recruited when humans plan actions (Lindner et al., 2010). Specifically, from an ACH perspective, we predict that M1 areas are more tightly coupled with the action planning network when a decision actually involves the execution of an action compared with when no action is executed.

We designed a functional Magnetic Resonance Imaging (fMRI) study similar to previously published neurophysiological monkey studies (Cisek and Kalaska, 2010). In a two-choice reaction time task, participants were first shown predictive cues (i.e., task-relevant information) before the actual go-signal appeared. The cue could indicate either a specific action plan (e.g., you will need to press left at the go-signal), a general action plan (i.e., the go-signal will indicate a left or right press, but you don’t know which one it will be) or could indicate that no action would be needed (e.g., the go-signal will indicate not to press).

According to our first hypothesis, if task-relevant information leaks up to M1 before movement onset, predictive cues should modulate M1 activity when no response is yet given. When cues are fully predictive (i.e., specific action plan), that is when they specify whether participants will have to press either the left or the right button press, contralateral M1 activity is expected to be higher than ipsilateral M1 activity. When a general action plan is specified, i.e., when both button presses are equally likely to be executed, activity in both M1 cortices is expected to be equal. Similarly, when no action plan is specified, we expect similar activity levels in both M1 cortices. Indeed, using the go-no-go task, Freeman and Aron have shown that when no action plan is required (i.e., no-go trial), cue-evoked CSE initially increases at similar levels compared to when an action is required (i.e., go trial; Freeman and Aron, 2016). Hence, sensorimotor cortex activity may display similar levels of activations when planning to act and not to act; similar results were obtained when humans planned what to reach for or what to avoid reaching (Lindner et al., 2010). According to our second hypothesis, we predicted stronger connectivity of M1 to the action planning network (i.e., fronto-parietal areas) when agents actually plan a general action (before movement onset). Such a prediction follows because planning a general action involves a competition between the afforded action plans, contrary to planning no action. Thus, if the competition reaches the M1, functional connectivity of M1 with the fronto-parietal areas (also coding for competing action plans) should be significantly stronger when planning a general action compared with planning no action. Similarly, functional connectivity should be stronger when planning a general action compared with planning a specific action. Indeed, competition between potential actions is higher in general than specific action planning.

## Materials and Methods

### Subjects

Sixteen right-handed participants (8 females; *M* = 24.6 years, *SD* = ± 3.22) with normal vision participated in this study approved by the local ethics committee (Comité d’Ethique Hospitalo-Facultaire Erasme-ULB, Brussels, Belgium). Participants received monetary compensation and provided written informed consent. One participant was excluded from the analyses after observing he moved his fingers during cue presentation (see below).

### Experimental Design

**Figure 1:**
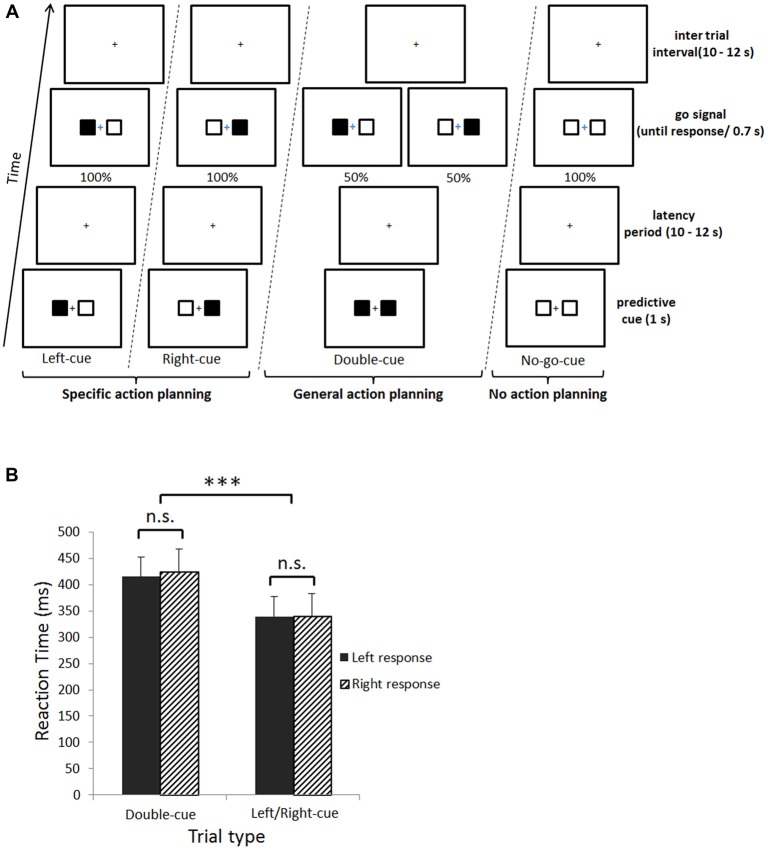
**(A)** Experimental design. Subjects were cued with predictive information associated with each upcoming button press. This cue could either be a left- or right-cue (i.e., specific action planning), a double-cue (i.e., general action planning), or a no-go-cue (i.e., planning to not act). Subsequently, there was a jittered latency period, followed by the go signal indicating subjects which button to press (Left-cue, Right-cue and Double-cue) or not (No-go-cue).** (B)** Behavioral results. Main effect of trial type for reaction times (RTs; ***p < 0.001). The X axis represents trial type, plain black fill are left button presses and upward diagonal black fill are right button presses. The Y axis represents mean RTs. Error bars denote the standard error of the mean and n.s. = non-significant difference.

Figure 1A illustrates the experimental design. Each trial started with the presentation of a predictive cue (1 s). The predictive cue was followed by a jittered fixation cross latency period (LP; 10–12 s). Subsequently, a go signal appeared indicating either to press the left or right button as fast as possible (until a response was given), or to passively watch the fixation cross (see below). Finally, a jittered inter trial interval (ITI) fixation cross was presented (10–12 s). The four predictive cues and three go signals were created by combining black and/or white squares presented left and right to a fixation cross. First, if the predictive cue was composed of left-black/right-white squares, participants were instructed to make a left upcoming button press at the go signal, composed of exactly the same pattern of squares (see left-cue trial in Figure 1A). Second, if the predictive cue was made of left-white/right-black squares, a right upcoming button press was required at the go signal, again made of the same pattern of squares (see right-cue trial in Figure 1A). Third, if the predictive cue was composed of two black squares, there were equal chances that subjects would have to make an upcoming left or right button press at the go signal (see double-cue trial in Figure 1A). In other words, the predictive double-cue could either be followed by a right-black or left-black go-signal; with a 0.5 probability of occurrence for each of these go signal stimuli. Fourth, if the predictive cue was made of two white squares, participants knew they would not have to make an upcoming button press at the go signal, which would also be composed of two white squares (see no-go-cue trial in Figure 1A). For this last trial type, participants had to passively fixate the cross during 700 ms in order to keep visual stimulation similar across trials. In sum, left/right-cue, double-cue and no-go-cue trials correspond respectively to planning a specific action, a general action and planning to not act. Participants were instructed to fixate the central cross during the entire trial and not to move unless for response presses (visually checked online, see below). To prevent confusion between predictive cues and go signals, the go signal fixation cross was presented in blue. The experiment was divided in six blocks of 20 trials (total of 120 trials), and the four trial types were randomly interleaved.

Long ITIs and LPs are important features in this slow event-related fMRI design. We used a long ITI because previous work demonstrated that the same cortical network (with similar levels of activation) is recruited whether one is planning an action or planning not to act (Kühn et al., 2009; Kühn and Brass, 2010). Consequently, we contrasted each condition to the ITI period. We used a long LP because our main question was whether task-relevant information before movement onset already biases M1 activity. We thus needed a design ensuring signal separation between cue events and motor responses. Although in theory (i.e., under the linear combination assumption; Dale and Buckner, 1997) this could be implemented through fast-event related design (but see Monti, 2011), in practice we can improve signal separation by lengthening the time between events. Because this aspect is crucial, we implemented a design with 10–12 s between events, combining both efficiency (i.e., compared to a 15–20 s LP) and good signal separation (i.e., compared to a much faster event-related design).

The experiment was implemented in Matlab 7.9 (MathWorks) using the Psychophysics Toolbox (Kleiner et al., 2007). Stimuli were projected on a translucent screen, and seen through a mirror mounted on the head coil. Responses were collected with a MRI-compatible response box (Current Designs).

### Eye Movement Recording

To exclude eye-movement related artifacts, eye movements were monitored using a MRI-compatible infrared eye tracking system (ASL ET-6000, Bedford, MA, USA). Gaze X and Y position coordinates and eye blinks were recorded during cue presentation and averaged across conditions. Subsequently, repeated-measures ANOVAs with trial type as single factor containing four levels (i.e., each trial type) were run separately on horizontal and vertical mean gaze positions as well as mean number of eye blinks.

### fMRI Data Acquisition Parameters

Data were acquired with a Philips Achieva 3-T (Philips Medical Systems, Best, Netherlands) scanner using a T2*sensitive gradient echo (EPI) sequence (TR = 2130 ms, TE = 40 ms, flip angle (FA) = 90°, SENSE acceleration factor 2.5, matrix size: 64 × 64 × 32; voxel size: 3.06 × 3.06 × 3 mm^3^; 32 transvers slices). Anatomical images were obtained using a T1-weighted sagittal 3D TFE sequence (TR = 1960 ms, TE = 4.60 ms, TI = 1040 ms, FA = 8°, FOV: 250 × 250 mm^2^, matrix size: 320 × 320 × 160, interpolated voxel size: 0.78 × 0.78 × 1.0 mm). The MR scanner was equipped with the Quasar imaging gradients (maximum amplitude and slew rate: 30 mT/m and 200 mT/m/ms) and an eight-channel SENSE head coil.

### fMRI Data Analysis

SPM 8 (Wellcome Trust Centre for Neuroimaging, London, UK) was used for pre-processing and fMRI data analysis. The first five volumes were discarded to avoid transient spin saturation effects. Individual pre-processing included adjustment for movement-related effects, spatial realignment of all functional images to the mean image, co-registration of the structural image with the mean realigned functional image, spatial normalization into standard stereotactic MNI space and spatial smoothing using a Gaussian kernel of 8 mm full width at half maximum (FWHM).

We first modeled each participant’s data with a general linear model (GLM) using an event-related approach. Regressors were convolved with the canonical hemodynamic response. The cut-off period for high-pass filtering was 128 s. Regressors of interest corresponded to the different predictive cues and button press events (delta functions). Six movement parameters derived from spatial realignment, and the latency period locked at predictive cue offset (boxcar function) were included as covariates of no interest in the design matrix. Effects of interest were tested by linear contrasts, generating statistical parametric maps [SPM(T)s]. Summary statistic images were entered in a second-level analysis in which subjects were treated as random effects (RFX). First, as a manipulation check, we mapped cue-related activity for each condition, as well as consistent cue-evoked activity (i.e., independent of the condition) across all participants. Then, we computed left > right button press and right > left button press contrasts. This allowed determining two functional ROIs associated with each button press at the group level. Next, we took the conjunction (overlap) between our functional ROIs and the Human Motor Area Template (HMAT) M1 area (Mayka et al., 2006); these conjunctions are from now on termed motor ROIs. Crucially, this allowed us to separate motor from premotor activity. To test our first prediction, namely the influence of task-relevant information on M1 before movement onset, we extracted beta weights from the motor ROIs (i.e., average within each motor ROI) during the left-cue and right-cue periods for each subject and each condition using the SPM(T)s generated at first level. Using STATISTICA 8.0, we then performed a 2 (motor ROI; left and right motor area) × 2 (trial type; left-cue, right-cue) repeated measures ANOVA on mean beta weights from the predictive cue period. We performed second, more exploratory analysis to investigate the relative increase/decrease in activity between specific action planning (left/right-cue trials), general action planning (double-cue trials), and planning to not act (no-go-cue trials). For this purpose, we performed a 2 (motor ROI; left and right motor area) × 4 (trial type; left-cue, right-cue, double-cue, no-go-cue) repeated measures ANOVA on mean beta weights from the predictive cue period.

To assess our second prediction that connectivity is fundamentally different when planning a general action and when planning to not act, we next carried out PsychoPhysiological Interaction (PPI) analyses (Gitelman et al., 2003). In PPI, the BOLD signal of one region (seed) is introduced as a regressor in the (first-level) GLM analysis. In addition, a condition regressor and a seed-by-condition interaction regressor are also included. Areas that differentially correlate with the seed in one condition compared with another condition will be identified by a significant seed-by-condition interaction regressor. Specifically, our PPI analyses may reveal brain networks in which activity after cue presentation but before movement onset is more tightly coupled with activity in the source M1 areas in the go compared with the no-go trials. Seeds of interest (SOI) were determined based on the response contrasts (i.e., left > right and right > left) and motor ROIs described in the previous paragraph. In particular, we compute two SOI (i.e., left and right) that corresponded to a 6 mm radius sphere around the left and right motor ROIs’ peak activation. For the first-level PPI-GLM, for each subject and each SOI, we computed the first eigenvariate of the extracted BOLD signal. Six independent new PPI-GLMs (one for each contrast) were then generated at the individual level, using the BOLD signal extracted at each SOI, the condition contrast vectors, and the seed-by-condition interaction regressors. The first two PPI-GLMs corresponded to the double-cue > no-go-cue and no-go-cue > double-cue contrasts, and were associated to our main second prediction. In line with our hypothesis, these contrasts should respectively reveal fronto-parietal connectivity and not reveal any fronto-parietal connectivity. Four additional exploratory PPI-GLMs corresponding to the left-cue > no-go-cue, right-cue > no-go-cue, double-cue > left-cue, and double-cue > right-cue contrasts, were used to investigate potential differences in connectivity between conditions. Finally, individual summary statistic images obtained at the first-level analysis were entered into a second-level (RFX) analysis using one-sample *t*-tests to test for group-level condition-specific effects.

## Results

The results reported below include only correct trials. Average accuracy was high (96.2 ± 2.65%).

### Behavioral Results

A 2 (trial type; double-cue, left/right-cue) × 2 (response side; left, right) repeated measures ANOVA on mean reaction time (RT) revealed a main effect of trial type (*F*_(1,14)_ = 189.8, *p* < 0.001). Double-cue trials induced slower RTs compared to left/right-cue trials (*M* = 420.2 ms, *SD* = ±67.8 and *M* = 339.8 ms, *SD* = ±62.7, respectively; Figure 1B). No significant interaction between trial type and response side was observed.

Mean pupil positions revealed no significant differences in eye movements across conditions (horizontal, *F*_(3,42)_ = 0.179, *p* = 0.910; vertical, *F*_(3,42)_ = 1.375, *p* = 0.263). Moreover, no significant differences in number of eye blinks were observed (*F*_(3,42)_ = 0.796, *p* = 0.503).

### fMRI Results

**Figure 2:**
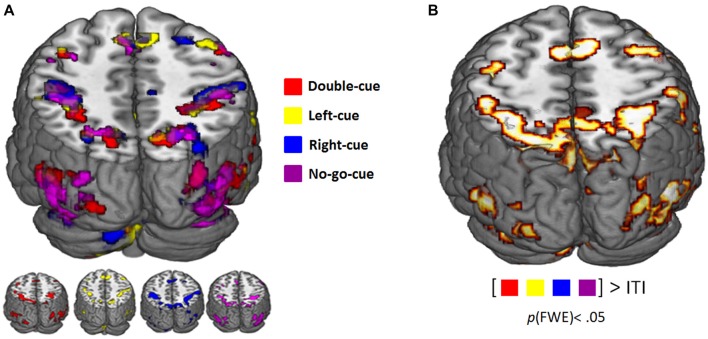
**(A)** Functional magnetic resonance imaging (fMRI) activity related to the presentation of the predictive cues (*p*^FWE-corrected^ < 0.05). Color coding corresponds to the different trial types (see legend). **(B)** Consistent cue-evoked activation (relative to implicit inter trial interval (ITI) baseline; *p*^FWE-corrected^ < 0.05) in superior parietal lobes (SPL), dorsal premotor cortex (PMd), supplementary motor area (SMA), middle frontal gyrus (MFG), inferior frontal gyrus (IFG), occipital areas, cerebellum across all participants.

Table 1 summarizes the activation clusters (FWE-corrected) of the relevant whole-brain contrasts described in this section. Figure 2A depicts whole-brain predictive cue-related activation for every trial type contrasted with implicit baseline (i.e., ITI). Regardless of trial type, predictive cues consistently elicit similar activity patterns. Furthermore, a linear combination of all cue-related activity against implicit baseline (ITI) evidenced a network of regions that showed significant cue-evoked activity in each individual subject (see Lindner et al., 2010). Bilateral Superior Parietal Lobes (SPL), Intraparietal Sulcus (IPS), dorsal premotor cortex (PMd), Supplementary Motor Area (SMA), and occipital areas were consistently activated across participants (Figure 2B).

**Table 1:** Summary of the activation clusters in the whole brain contrasts.

Contrast area	Local maxima MNI coordinates	Cluster size	Peak T	Cluster-level p(FEW-corr)
Left > Right button press (*)				
Right M1	40 −4 62	287	6.60	0.000
Right > Left button press (*)				
Left M1	−44 −32 58	334	7.22	0.000
All predictive cues > ITI (**)				
Superior parietal lobe L/R	46 −34 42	4660	19.65	0.000
Left cerebellum	−8 −82 −26	3633	17.35	0.000
Supplementary motor area	−8 8 56	801	16.52	0.000
Right inferior frontal gyrus	58 16 12	705	16.02	0.000
Left middle temporal gyrus	−46 −70 12	478	15.99	0.000
Right cerebellum	48 −56 −36	2157	14.04	0.000
Dorsal premotor cortex	34 8 62	265	13.30	0.000
Left middle frontal gyrus	−32 54 24	213	12.61	0.000
Right prefrontal cortex	28 42 20	248	11.56	0.000
**PPI**				
Double-cue > No-go-cue (*)				
**Left SOI**				
Right middle frontal gyrus	38 48 24	1804	9.34	0.000
Left parietal cortex	−26 −74 12	274	9.06	0.000
Left middle frontal gyrus	−40 38 22	1211	8.86	0.000
Inferior parietal cortex	44 −74 38	290	8.70	0.000
Superior parietal lobe	4 −66 56	386	7.40	0.000
Right cerebellum	36 −86 −14	668	7.12	0.000
Left cerebellum	−46 −54 −32	352	6.97	0.000
Left hippocampus	−40 −14 −14	219	6.60	0.000
**Right SOI**				
Right orbitofrontal gyrus	32 52 −4	110	10.17	0.005
Left inferior temporal gyrus	−62 −52 −12	94	9.40	0.010
Right middle frontal gyrus	48 42 20	179	7.78	0.001
Right middle temporal gyrus	64 −8 −18	93	7.29	0.010
Right caudate nucleus	18 18 12	69	6.85	0.027
Right temporal pole	44 22 −16	96	6.40	0.009
Double-cue > Left-cue (*)				
**Left SOI**				
Right frontal gyrus				
Right superior parietal lobe	50 4 16	1147	10.55	0.000
Left inferior frontal gyrus	34 −62 34	335	8.70	0.000
Right inferior parietal cortex	−26 30 −6	261	8.48	0.000
Left middle frontal gyrus	42 −36 34	496	7.67	0.000
Left inferior parietal cortex	−38 36 8	269	6.87	0.000
**Right SOI**	−38 −32 38	230	6.60	0.000
Right orbitofrontal cortex	30 56 0	56	6.07	0.023

(*) voxel-level threshold *p* = 0.0001 uncorrected, (**) voxel-level threshold *p*(FEW-corr) = 0.05. Only clusters surviving an extent threshold *k* > 200 and a *p*(FEW-corr) < 0.05 are reported (except for Right SOIs PPI where *k* > 20). For areas including multiple local maxima, the highest local maximum is reported.

**Figure 3:**
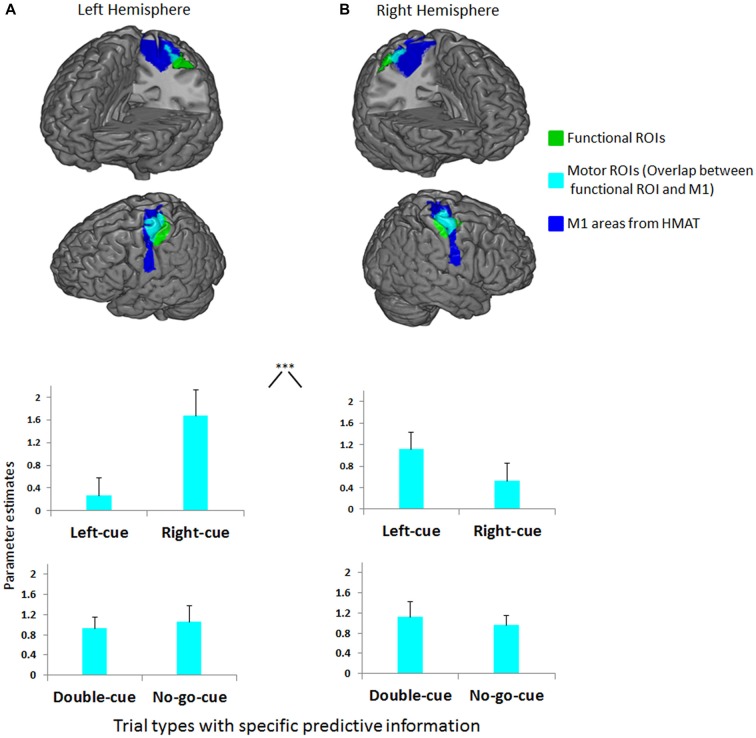
**(A)** Left functional ROI (green) and its overlap (cyan) with left primary motor cortex (M1) anatomical inclusive mask (blue). **(B)** Right functional ROI (green) and its overlap (cyan) with right M1 anatomical inclusive mask (blue). Depicted on both upper graphs are the mean parameter estimates of the left-cue and right-cue events extracted from both motor ROIs for each trial type. The graphs show a clear interaction between M1 side (left, right) and trial type (***p < 0.001). Activity before movement onset in contralateral M1 increases as the competition is biased in favor of a specific button press relative to activity in ipsilateral M1. Depicted on both lower graphs are the mean parameter estimates of the double-cue and no-go-cue events extracted from both motor ROIs for each trial type. No differences were observed between double-cue and no-go-cue elicited activity. Error bars on all graphs denote the standard error of the mean.

Analyses of response contrasts revealed two functional ROIs for right and left button presses (*p* < 0.0001 uncorrected; Figures 3A,B, respectively). The 2 (motor ROI location: left, right) × 2 (trial type: left-cue, right-cue) repeated measures ANOVA on cue-related activity revealed an interaction between motor ROI location and trial type (*F*_(1,14)_ = 27.8, *p* < 0.001). In accordance with our first prediction, as predictive information in favor of a specific button press increases, the evoked response in the contralateral M1 is increased relative to the ipsilateral M1 (Figures 3A,B). The second more exploratory ANOVA with factors motor ROI location (left, right) and trial type (left-cue, right-cue, double-cue) on cue-related activity also revealed an interaction between motor ROI location and trial type (*F*_(3,42)_ = 15.4, *p* < 0.001). As predicted (Kühn et al., 2009; Kühn and Brass, 2010; Lindner et al., 2010), the planned contrasts between the double-cue (i.e., general planning) and no-go-cue (i.e., no action planning) did not reveal a significant difference (*F*_(1,14)_ = 0.4, *p* = 0.537; see lower graphs in Figures 3A,B). To further investigate the relative increase/decrease in activity between each trial type, exploratory planned comparisons were performed. First, left/right-cue activity in ipsilateral motor ROIs was significantly lower than double-cue activity from both motor ROIs (*F*_(1,14)_ = 4.6, *p* < 0.05). Second, we did not observe a significant difference between left/right-cue activity in contralateral motor ROIs and double-cue activity from both motor ROIs (*F*_(1,14)_ = 1.17, *p* = 0.297). Note however that for this contrast (although not significantly) left/right-cue activity was higher than double-cue activity.

**Figure 4:**
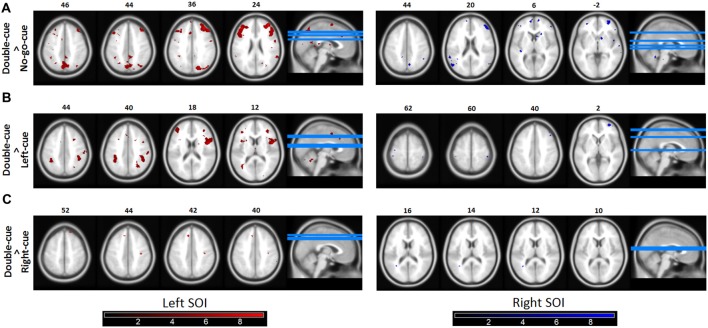
Functional connectivity (psychophysiological interactions, PPIs). **(A)** Brain areas in which activity is more tightly coupled with activity in the source motor cortex area in the double-cue event than in the no-go-cue event. Both the left and the right Seeds of interest (SOI) showed coupling with the fronto-parietal network. The left SOI was significantly coupled with the right and left MFG, SPL, and SMA. Similarly, the right SOI was coupled with the right and left MFG, left PMd, and SPL. **(B,C)** Similar as 4A for the contrasts double-cue > left-cue and double-cue > right-cue, respectively. As revealed in the figures, double-cue trials have a stronger coupling with the fronto-parietal network than left/right-cue trials. Note however that the coupling is decreased for the double-cue > right-cue trials. Functionally connected areas are displayed at *p^unc^* < 0.0001. Color bars represent *t*-statistic levels.

Concerning our second question, the functional connectivity patterns from each SOI (left and right; see fMRI data analysis for SOI definition) revealed a tighter coupling with parietal and frontal areas in the double-cue than in the no-go-cue trials (Figure 4A). Importantly, the opposite contrast (no-go-cue > double-cue) revealed no significantly connected areas (even at lowered threshold *p*^uncorrected^ < 0.001). These first two PPI contrasts confirm our prediction and suggest that although predictive (i.e., before movement onset) double and no-go cues elicit the same level of activations in both SOIs, the connected neural networks are fundamentally different.

The left/right-cue > no-go-cue contrasts revealed no significant coupling with parietal and frontal areas. To further test whether left/right-cue trials were less coupled with the fronto-parietal network than double-cue trials, we also performed double-cue > left/right-cue contrasts (Figures 4B,C). These exploratory PPI contrasts suggest that when there is less competition between action options, as for left-cue and right-cue trials, coupling of motor areas with the fronto-parietal network significantly diminishes.

## Discussion

In this fMRI experiment we manipulated planning (specific, general, and no action planning) before movement onset. In line with our first prediction, task-relevant information (i.e., biasing cues, presented long before movement onset) driving action selection is integrated up to M1. This confirms in humans the Thura and Cisek (2014) findings in monkeys that biased competition takes place up to M1. Moreover, our results revealed that planning a general action and planning to not act, are both active processes that elicit similar levels of activities in M1 (e.g., Kühn et al., 2009; Kühn and Brass, 2010). In line with our second prediction, although planning a general action and planning to not act induce equal levels of activation in M1, our PPI analyses showed tighter coupling with the fronto-parietal network in double-cue compared with no-go-cue trials (see Figure 4A). Crucially, this suggests that the M1 is not merely involved in executing a planned action but is actually part of the neural circuit subtending action planning. Indeed, although both trial types activate the fronto-parietal network (at predictive cue onset), cue-evoked activity extracted from our SOIs is more tightly coupled with fronto-parietal areas typically involved in action planning (Lindner et al., 2010; Gallivan et al., 2011b, 2016) when the experimental condition actually involves a response competition (i.e., the double-cue trials).

Our results are in line with several monkey neurophysiology studies showing the involvement of M1 areas in sensorimotor integration. For instance, in the work of Merchant et al. (2004a,b) monkeys had to perform a movement to intercept a moving stimulus. Prior to performing the movement, M1 neurons were activated by the moving stimuli. Their findings suggest that M1 is not merely involved in action execution, but also in action planning. Using a different approach, Gold and Shadlen (2000, 2003) observed similar findings in the oculomotor domain. These authors performed microstimulations to the frontal eye fields (FEF; i.e., the area responsible for eye movements) while monkeys carried out a random dot motion (RDM) task. Microstimulations evoked transient short eye movements; importantly, the deviation magnitude of these evoked eye movements depended on the evidence accumulated thus far. Hence, these results suggest that FEF are involved in the formation of the decision on top of planning and executing eye movements.

The tighter coupling with fronto-parietal areas during double-cue trials could occur because during these trials (i.e., when competition is high), participants oscillate between the two response options, producing correlated fluctuations between interconnected fronto-parietal regions. Such an explanation could also account for the decrease in coupling for left-cue and right-cue trials (see Figures 4B,C). Indeed, in left/right-cue trials, competition between action plans is rapidly biased in favor of a specific action (i.e., the competition is low). Furthermore, the PPI result showed a tighter coupling for the left than for the right M1 side. Although open for future research, this asymmetry in PPI results may be due to the fact that beta values were overall smaller in the right M1 (see Figure 3B; note that all participants were right-handed). In addition, this asymmetry cannot be caused by sex differences in neural activity patterns during visually guided actions (Gorbet and Sergio, 2007). Indeed, there was a close to equal split between the number of females and males in our sample.

Our result showing similar activation in M1 for planning a general action or planning not to act provides new methodological insights regarding the use of an appropriate motor control condition. Indeed, contrasting a general action plan to a condition instructing subjects not to plan an action (i.e., no-go-cues) may lead to misleading conclusions. Such a contrast would hide the activation associated with general action planning. One possible way around this issue is using a resting period control condition, as implemented in the present study. Another solution could be to make use of multi-voxel pattern analyses (MVPA). Indeed, although global activity in motor areas is similar for general action planning and planning to not act, several action planning studies using MVPA have shown that it is possible to decode (at the M1 level) prior to motor execution which type of movement (e.g., grasping, touching or reaching) will be executed (Gallivan et al., 2011a, 2013a,b, 2016). Therefore, instead of using classical contrast analyses, one can capitalize on the multivariate nature of the signal in multi-voxel analyses to investigate action planning. For instance, MVPA can measure the evolution of the action plans involved in the biased competition by revealing the temporal dynamics of decoding efficiency for distinct predictive cues (for a similar method in monkeys see Gail and Andersen, 2006).

In order to avoid misinterpretation, two methodological points related to our findings must be raised. First, participants fixated a central cross during cue presentation, like in monkey studies (Klaes et al., 2011). Indeed, overt attention modifies FEF activation (Premereur et al., 2015) that may in turn influence motor activity in the aforementioned neighboring ROIs recruited for button pressing. By controlling that gaze position and eye blinks during cue presentation were similar across conditions, we ensured that cue-related biased motor activity was not caused by overt spatial attention or eye-movement related artifacts (see Lindner et al., 2010; Kok et al., 2012). Also, cues (left and right squares) were presented close to the fixation point allowing participants to process cues without eye movements. Second, one may be concerned that activity from PMd leaked to M1 due to spatial smoothing or group averaging artifacts. However, a careful look at the ROIs in Figures 3A,B suggest that this is unlikely. The functional ROIs do not overlap with PMd, and if anything are actually posterior to the M1 anatomical mask. Furthermore, we exclusively looked at activation in the conjunction between the functional ROI and motor anatomical ROI. Together, these facts render it highly unlikely that our data is influenced by leakage of PMd activity. We can thus be confident that our data reflect M1 activity.

M1 dynamics showing higher activity for predictable upcoming button presses may account for faster RTs for upcoming actions. Indeed, a possible interpretation of our results is that biased competition operates as a push-and-pull mechanism leading to normalized activity, as in competitive networks (Wang, 2002; Cisek, 2006; Wong and Wang, 2006). In such networks, neuronal populations firing in favor of a specific button press compete through mutual inhibition, biased by the predictive cue. When participants plan for (or avoid) two equally probable upcoming button presses, the activity of each response option normalizes to half of the activity evoked by a single upcoming action plan. Similarly, when the task at hand involves three potential actions, activity normalizes to approximately a third of the original activity, and so on with an increasing number of potential actions. In line with this view, Praamstra et al. (2009) have shown that the amplitude of the lateralized readiness potential during movement planning was lowered as more potential actions were involved in the task at hand. However, when cues bias the competition for a specific action plan, then activity in favor of that action plan will increase whilst activity for the competing plan(s) will decrease. Hence, the response threshold will be attained faster for more predictable actions, yielding faster RTs. Such a competitive mechanism may take place through interhemispheric inhibitory connections between primary motor cortices (Duque et al., 2007). In value-based decision-making, this mechanism has been observed by Pastor-Bernier and Cisek (2011) when monkeys have to choose between two equally or unequally rewarded reaches (see also Bastian et al., 2003). This implies that the state of M1 activity is dynamical. This contrasts with the idea that the default state of M1 activity in our task is to represent two potential actions. If this was the case, we would not have observed different connectivity profiles between double-cue and no-go-cue trials. Furthermore, the difference in RT between double-cue and left/right-cue trials indicates that participants sustain action plan(s) in a manner that fits the abovementioned mechanism. Indeed, if participants had not maintained action plans during the long delay period, we would have expected RTs to be similar in both trial types.

Electroencephalography (EEG) studies suggest that when predictive information signals participants which of two competing button press is required, beta (15–30 Hz) synchrony between the ipsilateral M1 (i.e., EEG signals) and the non-selected hand response (i.e., electromyographic signals) is up-regulated prior to movement execution (van Wijk et al., 2009; see also Kilner et al., 2005). Hence, because previous research showed that such an up-regulation is also present when participants must withhold movement (Alegre et al., 2004; Zhang et al., 2008), it was recently suggested that increased corticospinal beta synchronization can bias the competition between potential actions by inhibiting the non-selected hand response (van Wijk et al., 2012).

Using a reaching task, we have recently shown that the state of the biased competition keeps evolving until very late in the reach movement (Calderon et al., 2015). In other words, the biased competition signals in M1 do not stop evolving after movement onset, but remain in constant evolution. Relatedly, Selen et al. (2012) provided evidence for a continuously evolving decision variable in the human motor system. In their study, participants had to judge the motion direction of a random dot display by moving a handle towards one of two target options. At different time points (i.e., different amounts of accumulated evidence) the arm of the participants was perturbed. The authors observed that the magnitude of reflex gains (i.e., muscle contraction responses when the muscle is stretched) correlated with the amount of evidence in favor of a specific evolving decision. In particular, as evidence in favor of a specific target option increased, so did the magnitude of reflex gains in the arm corresponding to that option. Thus, it was concluded that the motor system is recruited before a decision is completed. In the same vein, Donner et al. (2009) showed (using magnetoencephalography) that during a visual motion task in a random dot display, power in gamma and beta frequency ranges in the M1 could predict upcoming choices, i.e., report the presence or absence of motion. Specifically, as evidence accumulation unfolded throughout the trial, power in the beta/gamma frequency band dynamically decreased/increased, and the predictability of reporting the presence or absence of motion increased with evidence accumulation. In line with these results, we show that, compared to weaker information, stronger information in favor of a specific action induces higher/lower activity in the contralateral/ipsilateral M1. Altogether, these studies and the present work suggest that action selection is a continuous process that emerges from a biased competition up to M1.

Our data allow making tentative predictions for understanding neuropathological disorders. Indeed, one can predict that patients with lesions at the level of M1, or M1 efferent pathways, would demonstrate disturbed action planning abilities. Although our data do not strictly support that M1 is implicated in decision-making, this idea is defended in the ACH. Hence, under this view, lesions in M1 should also impair decision-making abilities. Obviously, patients with damaged to M1 regions do not demonstrate strong and obvious decision making deficits. Rather, the ACH implies that such patients may demonstrate subtler decision making inabilities, depending on the extent and severity of the lesion. Interestingly, this is exactly what was observed in recent work (Stewart et al., 2016) where stroke patients with lesions in efferent pathways of right M1 regions displayed a significantly lower proportion of correct visually guided action selections in comparison to age-matched control participants. However, the proportion of correct action selections in stroke patients was still far largely above chance level. Furthermore, Derosiere et al. (2015) instructed participants to perform button press decisions that were not merely based on perceptual processes, but also on value-based processes. Using continuous theta burst stimulation (cTBS) to disrupt M1 activity, they observed that participants’ valued-based decisions were not optimal (i.e., compared with control participants), when the left M1 was virtually lesioned (see also Zénon et al., 2015). These studies suggest the causal involvement of primary motor regions not only in action planning, but also perceptual and value-based decision-making. In sum, although the ACH is not clinically oriented, it may be used as a theoretical framework for understanding a series of neuropathological disorders and potentially implementing specific treatments by targeting the neural subsystems responsible for a given process within the decision making machinery.

It has been proposed that “embodied” decision making has an ecological advantage mainly in environments affording decisions between actions rather than abstract choices. In the wild, monkeys are prone to make primarily decisions between actions (e.g., what branch to jump to in order to escape a predator) rather than between abstract choices, unlike humans who may e.g., choose where to go on vacation. Thus, decision making in monkeys may ultimately be reduced to plan afforded actions and select the relevant one. Relatedly, as suggested by Rorie and Newsome (2005), “perhaps humans have evolved a more abstract decision-making module that is functionally separate from the motor effector systems that prepare and execute responses. For monkeys it may be the case that to see and decide is, in effect, to plan a motor response. For humans, on the other hand, the link between decision and action may well be more flexible, permitting longer lead times and more sophisticated processing between decision and action” (p. 43; see also Heekeren et al., 2004; Ho et al., 2009). In line with the ACH, our study suggests instead that cognitive information leaks up to M1. Hence, at the very least some dynamics proposed in the ACH apply to humans too. However, our study allows us to make such a statement for visually guided action planning. Whether more complex human decision-making involving reward probability (Yang and Shadlen, 2007; Kira et al., 2015) or preference judgments (Padoa-Schioppa and Assad, 2006), is based on the dynamics proposed in the ACH, remains open for future research (but see Derosiere et al., 2015).

Action planning and selection has mainly been tested within the domains of neuro-economics (i.e., choosing between two goods; e.g., Rangel and Clithero, 2012) and perceptual decision-making (for review see Heekeren et al., 2008; Forstmann et al., 2016). By means of contrasting events related to the presentation of goods or specific visual displays, fMRI studies from these domains have yielded clusters of activity in brain regions separate from those involved in implementing the action linked to the decision. In turn, it was suggested that decision-making stemmed from computations in these regions without considering the involvement of sensorimotor regions. Here, based on predictions of the ACH, we instead directly investigated the neural correlates of biased competition at the M1. We suggest that future studies should consider M1 involvement in human action planning and selection.

## Author Contributions

CBC, TV and WG designed the study. CBC collected and analyzed the data. CBC, FVO, PP, TV and WG wrote the article.

## Conflict of Interest Statement

The authors declare that the research was conducted in the absence of any commercial or financial relationships that could be construed as a potential conflict of interest.

## References

[B1] Alegre M. Gurtubay I. G. Labarga A. Iriarte J. Valencia M. Artieda J. (2004). Frontal and central oscillatory changes related to different aspects of the motor process: a study in go/no-go paradigms. Exp. Brain Res. 159, 14–22. 10.1007/s00221-004-1928-8 15480586

[B2] Bastian A. Schöner G. Riehle A. (2003). Preshaping and continuous evolution of motor cortical representations during movement preparation. Eur. J. Neurosci. 18, 2047–2058. 10.1046/j.1460-9568.2003.02906.x 14622238

[B3] Bolognini N. Ro T. (2010). Transcranial magnetic stimulation: disrupting neural activity to alter and assess brain function. J. Neurosci. 30, 9647–9650. 10.1523/JNEUROSCI.1990-10.2010 20660247 PMC6632835

[B4] Calderon B. C. Verguts T. Gevers W. (2015). Losing the boundary: cognition biases action well after action selection. J. Exp. Psychol. Gen. 144, 737–743. 10.1037/xge0000087 26076044

[B5] Cisek P. (2006). Integrated neural processes for defining potential actions and deciding between them: a computational model. J. Neurosci. 26, 9761–9770. 10.1523/JNEUROSCI.5605-05.2006 16988047 PMC6674435

[B6] Cisek P. (2007). Cortical mechanisms of action selection: the affordance competition hypothesis. Philos. Trans. R. Soc. Lond. B Biol. Sci. 362, 1585–1599. 10.1098/rstb.2007.2054 17428779 PMC2440773

[B7] Cisek P. Kalaska J. F. (2005). Neural correlates of reaching decisions in dorsal premotor cortex: specification of multiple direction choices and final selection of action. Neuron 45, 801–814. 10.1016/j.neuron.2005.01.027 15748854

[B8] Cisek P. Kalaska J. F. (2010). Neural mechanisms for interacting with a world full of action choices. Annu. Rev. Neurosci. 33, 269–298. 10.1146/annurev.neuro.051508.135409 20345247

[B9] Cisek P. Pastor-Bernier A. (2014). On the challenges and mechanisms of embodied decisions. Philos. Trans. R. Soc. Lond. B Biol. Sci. 369:20130479. 10.1098/rstb.2013.0479 25267821 PMC4186232

[B10] Coles M. G. H. Gratton G. Bashore T. R. Eriksen C. W. Donchin E. (1985). A psychophysiological investigation of the continuous flow model of human information processing. J. Exp. Psychol. Hum. Percept. Perform. 11, 529–553. 10.1037/0096-1523.11.5.529 2932529

[B11] Dale A. M. Buckner R. L. (1997). Selective averaging of rapidly presented individual trials using fMRI. Hum. Brain Mapp. 5, 329–340. 10.1002/(sici)1097-0193(1997)5:5<329::aid-hbm1>3.3.co;2-a 20408237

[B12] Derosiere G. Zénon A. Alamia A. Klein P.-A. Duque J. (2015). Contribution of primary motor cortex to perceptual and value-based decision processes. Front. Neurosci. 9:56 10.3389/conf.fnins.2015.89.0005625759638

[B14] Donner T. H. Siegel M. Fries P. Engel A. K. (2009). Buildup of choice-predictive activity in human motor cortex during perceptual decision making. Curr. Biol. 19, 1581–1585. 10.1016/j.cub.2009.07.066 19747828

[B15] Duque J. Murase N. Celnik P. Hummel F. Harris-Love M. Mazzocchio R.. (2007). Intermanual differences in movement-related interhemispheric inhibition. J. Cogn. Neurosci. 19, 204–213. 10.1162/jocn.2007.19.2.204 17280510

[B16] Forstmann B. U. Ratcliff R. Wagenmakers E.-J. (2016). Sequential sampling models in cognitive neuroscience: advantages, applications, and extensions. Annu. Rev. Psychol. 67, 641–666. 10.1146/annurev-psych-122414-033645 26393872 PMC5112760

[B17] Freeman S. M. Aron A. R. (2016). Withholding a reward-driven action: studies of the rise and fall of motor activation and the effect of cognitive depletion. J. Cogn. Neurosci. 28, 237–251. 10.1162/jocn_a_00893 26469745 PMC5208043

[B18] Gail A. Andersen R. A. (2006). Neural dynamics in monkey parietal reach region reflect context-specific sensorimotor transformations. J. Neurosci. 26, 9376–9384. 10.1523/JNEUROSCI.1570-06.2006 16971521 PMC6674591

[B19] Gallivan J. P. Chapman C. S. McLean D. A. Flanagan J. R. Culham J. C. (2013a). Activity patterns in the category-selective occipitotemporal cortex predict upcoming motor actions. Eur. J. Neurosci. 38, 2408–2424. 10.1111/ejn.12215 23581683

[B22] Gallivan J. P. McLean D. A. Valyear K. F. Culham J. C. (2013b). Decoding the neural mechanisms of human tool use. Elife 2:e00425. 10.7554/eLife.00425 23741616 PMC3667577

[B20] Gallivan J. P. Johnsrude I. S. Flanagan J. R. (2016). Planning ahead: object-directed sequential actions decoded from human frontoparietal and occipitotemporal networks. Cereb. Cortex 26, 708–730. 10.1093/cercor/bhu302 25576538 PMC4712801

[B21] Gallivan J. P. McLean D. A. Smith F. W. Culham J. C. (2011a). Decoding effector-dependent and effector-independent movement intentions from human parieto-frontal brain activity. J. Neurosci. 31, 17149–17168. 10.1523/JNEUROSCI.1058-11.2011 22114283 PMC6623835

[B23] Gallivan J. P. McLean D. A. Valyear K. F. Pettypiece C. E. Culham J. C. (2011b). Decoding action intentions from preparatory brain activity in human parieto-frontal networks. J. Neurosci. 31, 9599–9610. 10.1523/JNEUROSCI.0080-11.2011 21715625 PMC6623162

[B24] Gitelman D. R. Penny W. D. Ashburner J. Friston K. J. (2003). Modeling regional and psychophysiologic interactions in fMRI: the importance of hemodynamic deconvolution. Neuroimage 19, 200–207. 10.1016/s1053-8119(03)00058-2 12781739

[B25] Gold J. I. Shadlen M. N. (2000). Representation of a perceptual decision in developing occulomotor commands. Nature 404, 390–394. 10.1038/35006062 10746726

[B26] Gold J. I. Shadlen M. N. (2003). The influence of behavioral context on the representation of a perceptual decision in developing oculomotor commands. J. Neurosci. 23, 632–651. 12533623 10.1523/JNEUROSCI.23-02-00632.2003 PMC6741872

[B27] Gold J. I. Shadlen M. N. (2007). The neural basis of decision making. Annu. Rev. Neurosci. 30, 535–574. 10.1146/annurev.neuro.29.051605.113038 17600525

[B28] Gorbet D. J. Sergio L. E. (2007). Preliminary sex differences in human cortical BOLD fMRI activity during the preparation of increasingly complex visually guided movements. Eur. J. Neurosci. 25, 1228–1239. 10.1111/j.1460-9568.2007.05358.x 17331218

[B29] Gupta N. Aron A. R. (2011). Urges for food and money spill over into motor system excitability before action is taken. Eur. J. Neurosci. 33, 183–188. 10.1111/j.1460-9568.2010.07510.x 21091805 PMC4420634

[B31] Heekeren H. R. Marrett S. Bandettini P. A. Ungerleider L. G. (2004). A general mechanism for perceptual decision-making in the human brain. Nature 431, 859–862. 10.1038/nature02966 15483614

[B30] Heekeren H. R. Marrett S. Ungerleider L. G. (2008). The neural systems that mediate human perceptual decision making. Nat. Rev. Neurosci. 9, 467–479. 10.1038/nrn2374 18464792

[B32] Ho T. C. Brown S. Serences J. T. (2009). Domain general mechanisms of perceptual decision making in human cortex. J. Neurosci. 29, 8675–8687. 10.1523/JNEUROSCI.5984-08.2009 19587274 PMC2719543

[B33] Kilner J. M. Bott L. Posada A. (2005). Modulations in the degree of synchronization during ongoing oscillatory activity in the human brain. Eur. J. Neurosci. 21, 2547–2554. 10.1111/j.1460-9568.2005.04069.x 15932612

[B34] Kira S. Yang T. Shadlen M. N. (2015). A neural implementation of wald’s sequential probability ratio test. Neuron 85, 861–873. 10.1016/j.neuron.2015.01.007 25661183 PMC4365451

[B35] Klaes C. Westendorff S. Chakrabarti S. Gail A. (2011). Choosing goals, not rules: deciding among rule-based action plans. Neuron 70, 536–548. 10.1016/j.neuron.2011.02.053 21555078

[B36] Kleiner M. Brainard D. Pelli D. Ingling A. Murray R. Broussard C. (2007). What’s new in psychtoolbox-3? Perception 36, 1–16. Available online at: http://psychtoolbox.org/credits/

[B37] Klein-Flügge M. C. Bestmann S. (2012). Time-dependent changes in human corticospinal excitability reveal value-based competition for action during decision processing. J. Neurosci. 32, 8373–8382. 10.1523/JNEUROSCI.0270-12.2012 22699917 PMC3399779

[B38] Kok P. Rahnev D. Jehee J. F. M. Lau H. C. de Lange F. P. (2012). Attention reverses the effect of prediction in silencing sensory signals. Cereb. Cortex 22, 2197–2206. 10.1093/cercor/bhr310 22047964

[B39] Kühn S. Brass M. (2010). Planning not to do something: does intending not to do something activate associated sensory consequences? Cogn. Affect. Behav. Neurosci. 10, 454–459. 10.3758/cabn.10.4.454 21098806

[B40] Kühn S. Gevers W. Brass M. (2009). The neural correlates of intending not to do something. J. Neurophysiol. 101, 1913–1920. 10.1152/jn.90994.2008 19164107

[B41] Lindner A. Iyer A. Kagan I. Andersen R. A. (2010). Human posterior parietal cortex plans where to reach and what to avoid. J. Neurosci. 30, 11715–11725. 10.1523/JNEUROSCI.2849-09.2010 20810892 PMC2956133

[B42] Mayka M. A. Corcos D. M. Leurgans S. E. Vaillancourt D. E. (2006). Three-dimensional locations and boundaries of motor and premotor cortices as defined by functional brain imaging: a meta-analysis. Neuroimage 31, 1453–1474. 10.1016/j.neuroimage.2006.02.004 16571375 PMC2034289

[B43] Merchant H. Battaglia-Mayer A. Georgopoulos A. P. (2004a). Neural responses during interception of real and apparent circularly moving stimuli in motor cortex and area 7a. Cereb. Cortex 14, 314–331. 10.1093/cercor/bhg130 14754870

[B44] Merchant H. Battaglia-Mayer A. Georgopoulos A. P. (2004b). Neural responses in motor cortex and area 7a to real and apparent motion. Exp. Brain Res. 154, 291–307. 10.1007/s00221-003-1664-5 14579000

[B45] Michelet T. Duncan G. H. Cisek P. (2010). Response competition in the primary motor cortex: corticospinal excitability reflects response replacement during simple decisions. J. Neurophysiol. 104, 119–127. 10.1152/jn.00819.2009 20445034

[B46] Monti M. (2011). Statistical analysis of fMRI time-series: a critical review of the GLM approach. Front. Hum. Neurosci. 5:28. 10.3389/fnhum.2011.00028 21442013 PMC3062970

[B48] Padoa-Schioppa C. Assad J. A. (2006). Neurons in the orbitofrontal cortex encode economic value. Nature 441, 223–226. 10.1038/nature04676 16633341 PMC2630027

[B49] Pastor-Bernier A. Cisek P. (2011). Neural correlates of biased competition in premotor cortex. J. Neurosci. 31, 7083–7088. 10.1523/JNEUROSCI.5681-10.2011 21562270 PMC6703218

[B50] Praamstra P. Kourtis D. Nazarpour K. (2009). Simultaneous preparation of multiple potential movements: opposing effects of spatial proximity mediated by premotor and parietal cortex. J. Neurophysiol. 102, 2084–2095. 10.1152/jn.00413.2009 19657085 PMC6007848

[B51] Premereur E. Janssen P. Vanduffel W. (2015). Effector specificity in macaque frontal and parietal cortex. J. Neurosci. 35, 3446–3459. 10.1523/JNEUROSCI.3710-14.2015 25716844 PMC6605566

[B200] Rangel A. Clithero J. A. (2012). Value normalization in decision making: theory and evidence. Curr. Opin. Neurobiol. 22, 970–981. 10.1016/j.conb.2012.07.011 22939568 PMC4334383

[B52] Rorie A. E. Newsome W. T. (2005). A general mechanism for decision-making in the human brain? Trends Cogn. Sci. 9, 41–43. 10.1016/j.tics.2004.12.007 15668095

[B53] Selen L. P. J. Shadlen M. N. Wolpert D. M. (2012). Deliberation in the motor system: reflex gains track evolving evidence leading to a decision. J. Neurosci. 32, 2276–2286. 10.1523/JNEUROSCI.5273-11.2012 22396403 PMC3299561

[B54] Shadlen M. N. Kiani R. Hanks T. D. Churchland A. K. (2008). “Neurobiology of decision making: an intentional framework,” in Better than Conscious? Decision-Making, the Human Mind, and Implications for Institutions, eds Engel C. Singer W. (Cambridge, MA: MIT Press), 71–102.

[B55] Stewart J. C. Dewanjee P. Shariff U. Cramer S. C. (2016). Dorsal premotor activity and connectivity relate to action selection performance after stroke. Hum. Brain Mapp. 37, 1816–1830. 10.1002/hbm.23138 26876608 PMC4836996

[B57] Thura D. Beauregard-Racine J. Fradet C.-W. Cisek P. (2012). Decision making by urgency gating: theory and experimental support. J. Neurophysiol. 108, 2912–2930. 10.1152/jn.01071.2011 22993260

[B56] Thura D. Cisek P. (2014). Deliberation and commitment in the premotor and primary motor cortex during dynamic decision making. Neuron 81, 1401–1416. 10.1016/j.neuron.2014.01.031 24656257

[B58] van Wijk B. C. M. Beek P. J. Daffertshofer A. (2012). Neural synchrony within the motor system: what have we learned so far? Front. Hum. Neurosci. 6:252. 10.3389/fnhum.2012.00252 22969718 PMC3432872

[B59] van Wijk B. C. M. Daffertshofer A. Roach N. Praamstra P. (2009). A role of β oscillatory synchrony in biasing response competition? Cereb. Cortex 19, 1294–1302. 10.1093/cercor/bhn174 18836098

[B60] Wang X.-J. (2002). Probabilistic decision making by slow reverrberation in cortical circuits. Neuron 36, 955–968. 10.1016/s0896-6273(02)01092-9 12467598

[B61] Wong K.-F. Wang X.-J. (2006). A recurrent network mechanism of time integration in perceptual decisions. J. Neurosci. 26, 1314–1328. 10.1523/JNEUROSCI.3733-05.2006 16436619 PMC6674568

[B62] Yang T. Shadlen M. N. (2007). Probabilistic reasoning by neurons. Nature 447, 1075–1080. 10.1038/nature05852 17546027

[B63] Zénon A. Klein P. A. Alamia A. Boursoit F. Wilhelm E. Duque J. (2015). Increased reliance on value-based decision processes following motor cortex disruption. Brain Stimul. 8, 957–964. 10.1016/j.brs.2015.05.007 26279406

[B64] Zhang Y. Chen Y. Bressler S. L. Ding M. (2008). Response preparation and inhibition: the role of the cortical sensorimotor β rhythm. Neuroscience 156, 238–246. 10.1016/j.neuroscience.2008.06.061 18674598 PMC2684699

